# Temporo-Frontal Coherences and High-Frequency iEEG Responses during Spatial Navigation in Patients with Drug-Resistant Epilepsy

**DOI:** 10.3390/brainsci11020162

**Published:** 2021-01-26

**Authors:** Aljoscha Thomschewski, Eugen Trinka, Julia Jacobs

**Affiliations:** 1 Affiliated Centre of the European Reference Network EpiCARE, Department of Neurology and Centre for Cognitive Neuroscience, Christian-Doppler Medical Centre, Paracelsus Medical University, Ignaz-Harrer-Str. 79, 5020 Salzburg, Austria; e.trinka@salk.at; 2 Department of Psychology, Paris-Lodron University of Salzburg, Hellbrunnerstraße 34, 5020 Salzburg, Austria; 3 Member of the European Reference Network EpiCARE, Epilepsy Center, Medical Center, Faculty of Medicine, University of Freiburg, Breisacher Straße 64, 79106 Freiburg, Germany; Julia.Jacobs-LeVan@albertahealthservices.ca; 4 Department of Neuropediatrics and Muscle Disorders, University Hospital Freiburg, Mathildenstraße 1, 79106 Freiburg, Germany; 5 Room 293, Alberta Children’s Hospital Research Institute and Hotchkiss Brain Institute, University of Calgary, Heritage Medical Research Building, 3330 Hospital Dr. NW, Calgary, AB T2N 4N1, Canada

**Keywords:** high-frequency oscillations (HFOs), spatial navigation, stereo EEG, functional connectivity, EEG-coherence

## Abstract

The prefrontal cortex and hippocampus function in tight coordination during multiple cognitive processes. During spatial navigation, prefrontal neurons are linked to hippocampal theta oscillations, presumably in order to enhance communication. Hippocampal ripples have been suggested to reflect spatial memory processes. Whether prefrontal-hippocampal-interaction also takes place within the ripple band is unknown. This intracranial EEG study aimed to investigate whether ripple band coherences can also be used to show this communication. Twelve patients with epilepsy and intracranial EEG evaluation completed a virtual spatial navigation task. We calculated ordinary coherence between prefrontal and temporal electrodes during retrieval, re-encoding, and pre-task rest. Coherences were compared between the conditions via permutation testing. Additionally, ripples events were automatically detected and changes in occurrence rates were investigated excluding ripples on epileptic spikes. Ripple-band coherences yielded no general effect of the task on coherences across all patients. Furthermore, we did not find significant effects of task conditions on ripple rates. Subsequent analyses pointed to rather short periods of synchrony as opposed to general task-related changes in ripple-band coherence. Specifically designed tasks and adopted measures might be necessary in order to map these interactions in future studies.

## 1. Introduction

Throughout the last decades, EEG research has increasingly focused on frequencies above the gamma band range. The so-called high-frequency EEG components have predominantly gained interest as a surrogate marker for epileptogeneity (e.g., References [1,2,3]). In this context these oscillations have been studied extensively as distinctive events within the EEG signal, so-called high frequency oscillations (HFOs), defined as spontaneous oscillatory events of at least four periods in a frequency range from 80 to 500 Hz that distinctively stand out from the background signal [4]. Furthermore, HFOs are distinguished according to their frequencies and divided into the ‘slower’ ripples with frequencies between 80 and 250 Hz, fast ripples with frequencies between 250 and 500 Hz [5], and very fast ripples exceeding frequencies of 500 Hz [6,7].

While being most extensively researched in the context of epilepsy, where HFOs have been proposed as a possible surrogate marker for epileptogenesis (e.g., References [2,4,8]), in some brain regions HFOs have been observed in connection with cognitive processing, such as during sensorimotor [9,10,11], visual [12,13], or memory processes [14,15]. Especially, hippocampal HFO occurrences have been closely linked to memory consolidation in several studies with animals [16,17,18] and human subjects [14,15,19]. Kunii et al. [15] for instance used high frequency activity recorded in the parahippocampal gyri during a memory task to calculate a laterality quotient for memory dominance. They found epilepsy patients undergoing resective surgery on the so-defined memory-dominant side to experience subsequent memory decline [15]. Just recently, Pu et al. [19] recorded HFOs in the hippocampal region in healthy human subjects using magnetoencephalography during the inter-trial resting period of a virtual navigation task. Not only did they find HFOs, but the high frequency activity could further be linked to a subsequent performance improvement, suggesting these HFOs to reflect hippocampal reactivation [19]. Taking these findings into consideration, HFOs appear to be an interesting marker to document memory performance.

However, there have also been contradicting findings regarding the physiological meaning of spontaneously occurring ripples, especially when recorded in epilepsy patients [20]. Furthermore, the criteria for distinguishing physiologic and pathologic HFOs still remain disputed. This constitutes an ongoing challenge [21], especially since pathologic ripples might have a negative effect on cognition, as observed for other interictal epileptiform discharges (e.g., References [22,23,24]). One suggestion though has been that physiologic HFOs, unlike epilepsy-related HFOs, may appear less distinct from the background or even as continuous high frequency activity within the EEG signal [25,26]. This notion indicates that it might be worthwhile to consider not only single events, but also the high frequency band itself to reflect physiologic processes. Furthermore, it has been pointed out that coupling of ripples between the medial temporal lobe and association cortices might constitute an underlying mechanism of memory retrieval [27]. Taken these thoughts together with the idea of evoking physiologic HFOs by certain tasks, one approach to further investigate physiologic HFO activity may lie in considering connectivity measures derived from these high frequencies.

It is well established that the hippocampi are very much involved in memory and spatial navigation [28,29,30]. Besides them, prefrontal regions have also been identified to be immanent for successful encoding and retrieval of spatial contents [31,32,33]. During tasks, such as those involving spatial navigation, prefrontal neurons lock to the hippocampal theta oscillations, presumably reflecting communication between both structures [34,35,36]. Building on the presumed prefrontal-hippocampal-interaction, we investigated the connectivity between these sites in the invasive EEG of patients performing a virtual-reality-based spatial navigation task. Based on the existing literature we expected to find increased connectivity between hippocampal and prefrontal regions during retrieval and re-encoding of spatial information, not only in measures derived from the theta and gamma band [37,38,39], but also from the ripple-band. In addition, we investigated whether ripple event occurrence rates changed throughout the task, and whether changes in both the event rates and the high-frequency derived connectivity measure could be associated similarly to cognitive processes underlying the task.

## 2. Materials and Methods

### 2.1. Participants

Subjects were recruited amongst epilepsy patients who were undergoing invasive EEG recordings for presurgical evaluation in three epilepsy centers: Beijing (CN), Bielefeld (GER), and Freiburg (GER). In order to explore the prefrontal-hippocampal interaction we only included patients, who had invasive recordings from both within the frontal lobe and the hippocampal region. Since March 2017, a total of 27 patients had been recruited to participate in the study. Based on the iEEG electrodes’ localization, we were able to identify 12 patients suitable to be included into this analysis. The sample’s age ranged from to 19 to 33 years (mean age = 25.17, SD = 4.09) and six included patients (50%) were female. All patients were implanted with iEEG electrodes that amounted to an average of 118 recorded channels (between 50 and 192). Written informed consent was obtained from all subjects prior to the experiment and ethical approval was received at all three locations prior to enrollment.

### 2.2. Experimental Design

The paradigm was adapted from Doeller et al. [40] and has been described previously [41,42,43]. While being under continuous video-EEG-monitoring for diagnostic purposes, participating patients performed a cue-location memory task navigating freely in a circular virtual arena. The virtual arena comprised a grassy plane (diameter of 9500 virtual units) bounded by a cylindrical cliff. Patients completed the task on a laptop using the arrow keys for moving forward, turning left and right, and the spacebar to indicate their response.

At the very beginning of the first session, participants collected eight everyday objects (randomly drawn from a total number of 12 potential objects) from different locations in the arena (“initial learning phase”). This time period (variable duration of approximately two minutes, as the whole task was self-paced) was excluded from all analyses. Several constraints ensured that the object positions were not too close to the cylindrical cliff, the center, and other objects. Afterwards, participants completed a variable numbers of trials, depending on individual movement speed. Each trial consisted of four different phases.

First, one of the eight cues was presented for 2 s. Afterwards, participants were asked to navigate to the assumed corresponding location within the virtual environment (“retrieval”). During the retrieval period, the cue was not presented anymore but had to be kept in mind by the subject. After participants had indicated their response via a button press, they received feedback depending on response accuracy. Finally, the cue was presented at the correct location and participants had to collect the cue, allowing them to gradually improve their memory for the cue-location associations (“re-encoding”). After each trial, a fixation crosshair cursor was shown for a variable duration of 3 to 5 s (uniformly distributed).

To align behavioral and EEG data, experimental triggers were either detected using a phototransistor attached to the screen, marking onsets and offsets of the cue-phase, or using an independent custom MATLAB program that sent triggers both to the paradigm and to the EEG recording software with randomly jittered intervals between 0.5 and 5 s. Patients were asked to complete up to 160 trials but were instructed to pause or quit the task whenever they wanted. Experimental events were written to a log file.

### 2.3. iEEG Analyses

All included patients had been implanted with intracranial stereo EEG electrodes for purposes of pre-surgical evaluation. Sampling rates for iEEG data were 2000 Hz except for patient 3, whose recording was sampled at 1000 Hz. All iEEG data were visually inspected to exclude channels with bad signal quality due to either technical or material issues. Further, only iEEG channels situated within the grey matter and in the regions of interest were considered for analysis.

To identify the channel locations, individual MRI and CT scans served as reference. For patients recorded in Freiburg or Bielefeld, pre- and post-implantation MRIs were co-registered, skull-stripped, and normalized to MNI space using FSL (https://fsl.fmrib.ox.ac.uk/fsl/fslwiki/FSL). Then, we used PyLocator (http://pylocator.thorstenkranz.de/) in order to manually mark channels within the normalized post-implantation images. Channels recorded in patients from Beijing were localized to MNI space via pre-implantation MRI and post-implantation CT scans, using a custom toolbox [44]. Within FreeSurfer, (https://surfer.nmr.mgh.harvard.edu/) cortical/subcortical labels were assigned to each MNI coordinate of the average structural template from FSL. Based on these labels the individual channel coordinates were categorized according to the closest cortical/subcortical labels, enabling the identification of the respective regions of interest for each patient’s iEEG channels.

EEG analysis comprised two different strategies. (i) An analysis of ripple event rates during the different experimental conditions and (ii) an analysis of inter-channel coherence. On the basis of the assigned closest cortical/subcortical regions, we grouped the implanted channels within the frontal and temporal lobes, in order to align sample sizes between patients for the subsequent coherence analyses. Six regions of interest were predefined this way: inferior-frontal, medial-orbitofrontal, superior-frontal, and lateral-orbitofrontal within in the frontal lobe, as well as hippocampus and parahippocampus as temporal regions. As the iEEG channels were differently referenced across the three centers, we re-referenced all iEEG channels to a common average reference before further analyses, as suggested by previous studies using a similar approach [45]. All iEEG analyses were performed using MATLAB (release R2018b, The Mathworks, Natick, MA, USA).

#### 2.3.1. Ripple Event Analysis

For event analysis, we used the code packages contained in MOSSDET, a software developed by Reference [46], for automatic detection of EEG elements. MOSSDET is based on kernelized support-vector-machines (SVM) and enables not only the detection of HFOs, but also of epileptiform spikes in the data. Using the implemented algorithms we detected ripples and ripples-on-spikes in each patient. The criteria for ripple detection implemented within the algorithm defined ripples as events of not longer than 250 ms that contained at least four consecutive oscillations within the respective frequencies. The classification of selected events was based on several features derived from the Morlet Wavelet Transform of 25 ms-long overlapping epochs, including power, line length, amplitude range, symmetry, mobility, and complexity [46].

Detected events were exported as a .csv file, and the number of events were counted for each retrieval and re-encoding trial, as well as the resting phase before. The absolute values were then divided by the number of channels and time samples for each condition in order to receive one relative index of ripple and ripple-on-spike occurrence rates for each participant and condition. Event occurrence rates were compared between the three conditions (resting, retrieval, and re-encoding), as well as the two main regions of interest (frontal, and (para-)hippocampal), using a repeated measures ANOVA in SPSS Statistics (Version 21; IBM Corp.; Armonk, NY, USA). Condition, event-type (ripple or ripple-on-spike) and region of interest were entered as within-subject factors. As dependent variable we entered the event rate per condition relative to the conditions’ duration and number of channels per main region. Mauchly’s test for sphericity suggested a violation of unequal variances for event rates across conditions only ($\chi_{\left(2\right)}^{2}$ = 8.9, *p* = 0.012), so we adjusted the degrees of freedom using the Greenhouse-Geisser method.

#### 2.3.2. iEEG Connectivity Analysis

For the further iEEG analysis, we first segmented the obtained signals according to the experimental log files. Retrieval and re-encoding phases were separated and the resting period before the virtual reality task was segmented into equally sized segments for each participant. Second, autoregressive models were calculated for each segment using the mvfreqz.m and mvar.m functions implemented within the BioSig toolbox [47]. A multivariate autoregressive model was calculated for all channel x channel combinations and with a model order of 474, chosen in order to account for the high number of frequency components. For each calculation with the given model order, we excluded segments that were too short for the suggested ratio of 3:1 between given samples and the number of estimates [48]. The resting period segment sizes were individually fixed beforehand in order to adhere to this ratio.

From the results we derived real valued coherence, an undirected measure of connectivity, which is taking into account only the real part of the complex coherence [49] and which has been suggested to be a reliable (in terms of stability over time) marker in neurological conditions, such as epilepsy [50]. Both measures were computed for 1 Hz frequency steps between 1 and 249 Hz. Frequencies within the line noise range, as well as their exponential harmonics, were not considered. The coherence measures were then averaged according to the respective frequency bands of interest: theta from 4 to 7 Hz [51], gamma from 40 to 79 Hz, and the ripple band from 80 to 249 Hz. The resulting coherence-matrices for each trial of the three conditions and for each patient were then statistically analyzed.

In order to help interpret the coherence results we additionally compared the frequency contents derived from each subject’s iEEG signal between the three conditions (retrieval vs. resting, re-encoding vs. resting, retrieval vs. re-encoding). For this purpose, we Fourier-transformed the signals of each segment for frequencies between 1 and 250 Hz. Subsequently, the so-obtained spectra for each iEEG channel were averaged according to the channels’ locations, allowing for a group-comparison later on. Finally, the spectra were averaged across the single trials for each subject.

For the assessment of possible changes in the inter-channel connectivity, we compared markers derived from experimental conditions’ EEG segments with the resting EEG coherence using permutation t-tests for each patient individually. Permutation testing was performed using the permtest.m function in MATLAB [52] with 1000 iterations. The result is a matrix of test statistics for each individual patient hinting to differences between the conditions and the resting segments for specific frequency bands and channel × channel pairs. Similarly, permutation testing was used to compare the averaged Fourier spectra of each patient between the three conditions resulting in test statistics that indicate differences between the conditions in all computed frequencies and the six brain regions of interest.

Absolute *p*-values were interpreted, though keeping in mind that due to family-wise error cumulation the alpha level would have to be adjusted when interpreting the results dichotomously as being significant or not. While the permutation testing was corrected for multiple comparisons (i.e. number of channel pairs and frequency steps), performed within each subject, we did not correct for the number of subjects. With one statistical test for the event analysis, three comparisons between the Fourier spectra, as well as 24 tests for the connectivity analyses (one test each for both conditions times 12 patients), a conservative correction using the Bonferroni method would result in a further adjusted *p*-threshold of 0.0018 (*p*/28; Reference [53]).

#### 2.3.3. Further iEEG Investigation

After retrieving the results from the described analyses, we decided to further investigate possible short-term changes in coherence that were not strictly locked to paradigm trials. For this purpose, we again calculated the autoregressive model as described above, but with a moving window of 47 s, a length chosen in order to adhere to the above-mentioned criteria. The autoregressive model and the extracted coherence measure were calculated for the entire iEEG recording with the window being shifted for one second (1000 or 2000 samples), in order to observe changes in coherences at a resolution of seconds. The resulting coherences can be viewed in a timeline taking into account the beginning of each trial’s conditions in the paradigm, indicating changes in interaction strengths between each channel pair and frequency band of interest.

## 3. Results

### 3.1. Ripple Event Analysis

Table 1 shows the number of channels within the six regions of interest, the overall ripple rates per second (for all regions), and the mean distance of the collected items’ recalled positions during retrieval from their actual positions within the virtual arena. The data do not suggest an effect of the task being performed on the ripple rates across the patients, also being reflected by the test results revealing no effect for condition ($F_{\left(1.26,13.84\right)}$ = 0.5, *p* = 0.533). Due to the small number and high variance in event rates we were only able to test an effect on the basis of average trials for each condition and not on a single-trial level.

**Figure 1:**
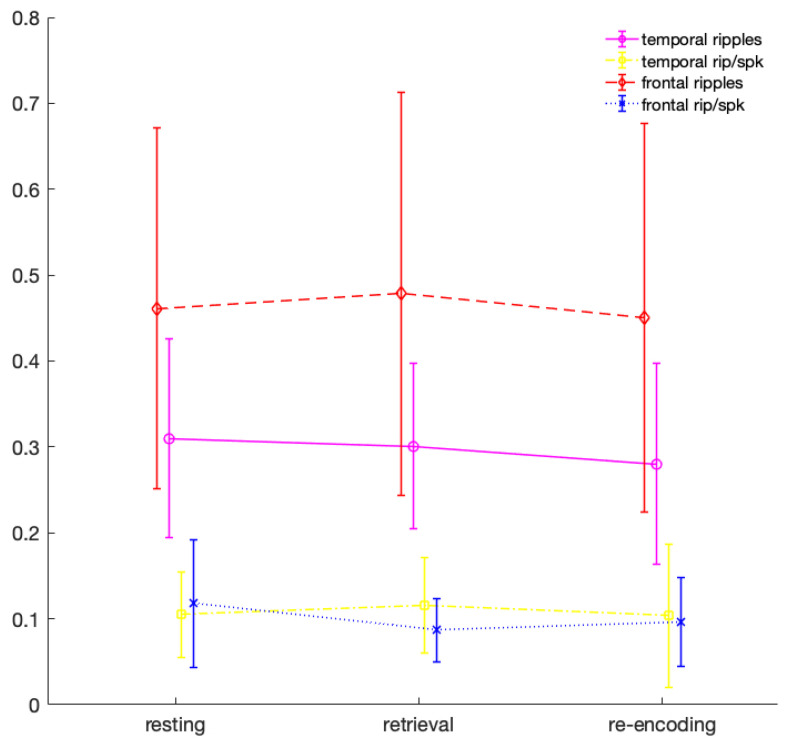
Mean ripple and ripple-on-spike (rip/spk) rates per second during the three conditions for frontal and temporal regions, relative to the conditions’ duration and each patients’ number of channels. Error bars represent 95%-confidence intervals.

Analysis, however, revealed lower event rates for ripples-on-spikes as compared to spikes ($F_{\left(1,11\right)}$ = 48.55, *p* <0.001), an effect that can also be appreciated when looking at the mean event rates (see Figure 1). Furthermore, analysis revealed a trend for an interaction between the region of interest and the type of measure ($F_{\left(1,11\right)}$ = 3.75, *p* = 0.079). However, no other interaction effects between the factors were revealed. What becomes apparent when considering event occurrence rates for each region of interest, is a high variability between the patients, with mean ripple rates between 0.171 and 0.266, and standard deviations between 0.046 and 0.19. Similar variations can be observed for ripples on spikes with means between 0.027 and 0.114, and standard deviations between 0.017 and 0.142. This variance between patients appears even more remarkable in the context of inter-channel coherence.

### 3.2. iEEG Coherence Results

iEEG analysis regarding connectivity did not reveal any general effects across patients. Table 2 shows the number of patients revealing statistically noticeable differences in coherences between task conditions and pre-task resting. It also indicates the number of patients with localized electrodes in each brain region pairs. As can be seen, only a proportion of patients revealed statistically significant differences in coherence matrices at all. Furthermore, some patients revealed higher coherences, while others elicited lower coherences during the task conditions in comparison to pre-task resting. This incongruence can be further appreciated when considering the spectral power densities derived from the conditions’ iEEG segments.

**Figure 2:**
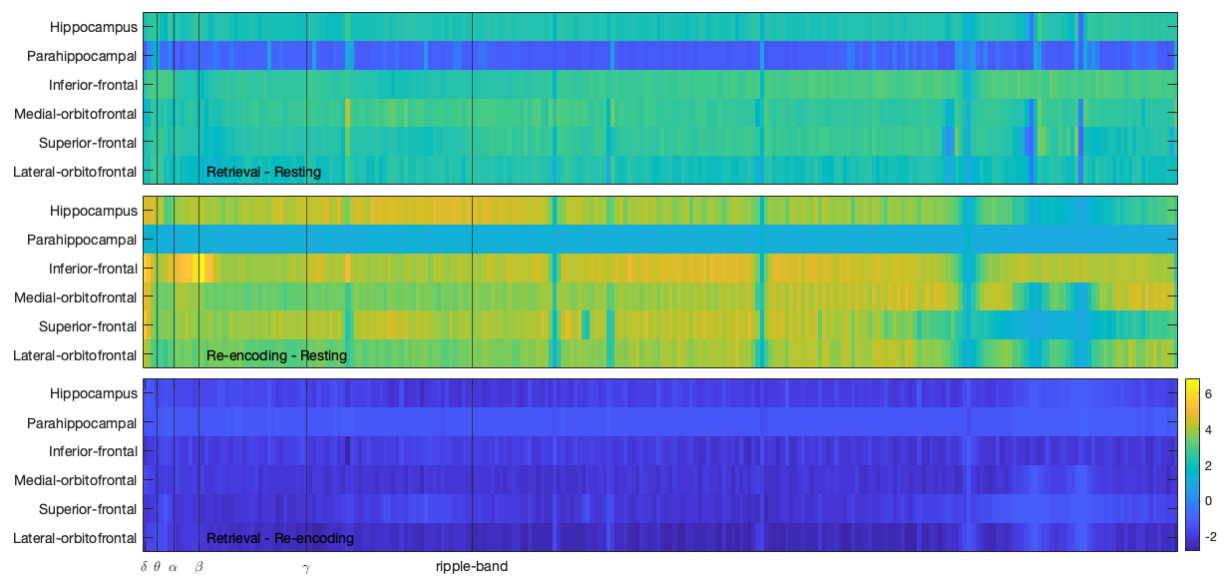
Test statistics for the comparisons between the Fourier spectra. The subfigures show one comparison each for the six brain regions of interest. The abscissae indicate the compared frequency bins from 1 to 250 Hz.

Figure 2 shows the test statistics for these comparisons, revealing no noticeable trends of differences between the power density spectra. Statistical significance testing concurred with this observation. Interestingly, the results suggest that parahippocampal regions are not greatly involved in the retrieval phase of the spatial navigation task. However, only one-third of the patients had electrodes implanted within this region. Furthermore, power spectra suggest higher task-involvement of all regions of interest during re-encoding than during retrieval when compared to the resting period.

**Figure 3:**
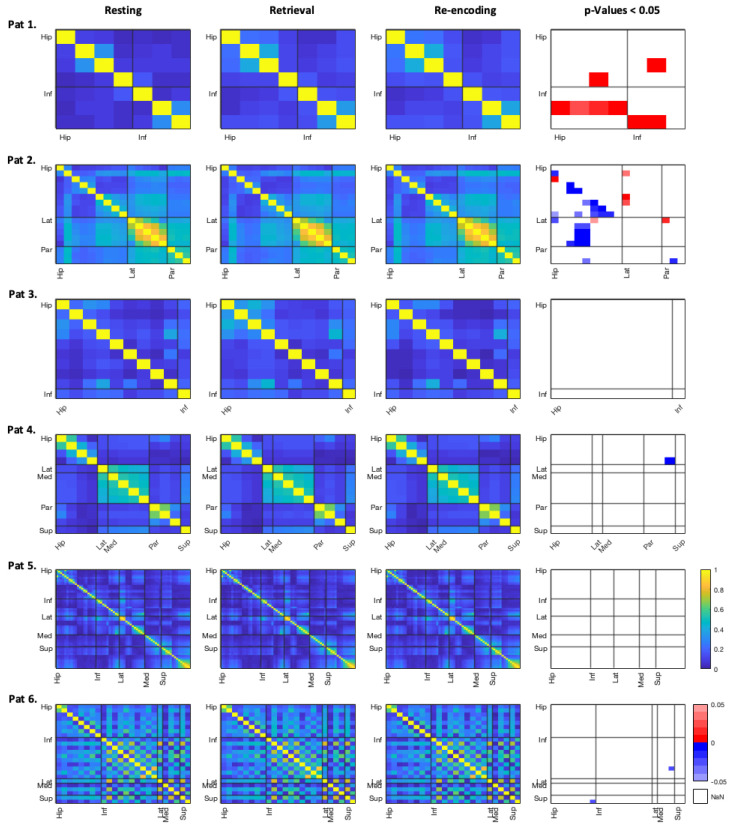
Coherences for patients 1–6 within the ripple band. Absolute coherence values in channel x channel matrices for both experimental conditions, as well as resting, are displayed (columns 1–3). Column 4 shows the respective contrasts between retrieval versus resting in the bottom left triangle under the diagonal, and re-encoding versus rest in the upper right triangle. Red (positive) *p*-values represent higher connectivity in the experimental condition, and blue (negative) values represent higher connectivity during the resting. Brain regions of interest are abbreviated as follows: Hip = hippocampus, Inf = inferior-frontal, Lat = lateral-orbitofrontal, Med = medial-orbitofrontal, Par = parahippocampus, Sup = superior-frontal.

**Figure 4:**
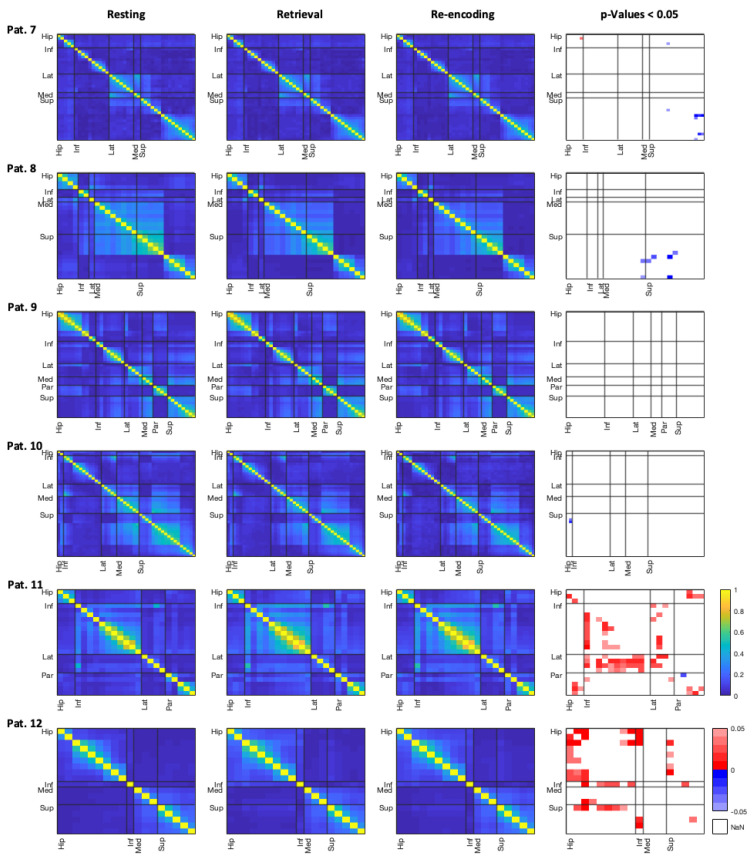
Coherences for patients 7–12 within the ripple band. Absolute coherence values in channel x channel matrices for both experimental conditions, as well as resting, are displayed (columns 1–3). Column 4 shows the respective contrasts between retrieval versus resting in the bottom left triangle under the diagonal, and re-encoding versus rest in the upper right triangle. Red (positive) *p*-values represent higher connectivity in the experimental condition, and blue (negative) values represent higher connectivity during the resting. Brain regions of interest are abbreviated as follows: Hip = hippocampus, Inf = inferior-frontal, Lat = lateral-orbitofrontal, Med = medial-orbitofrontal, Par = parahippocampus, Sup = superior-frontal.

In contrast, coherences during retrieval seem to differ more strikingly from resting than coherences during re-encoding. As can be seen in the individual coherence matrices for each single patient, most patients did not reveal a significant effect of different ripple-band coherences (see Figure 3 and Figure 4). Additionally, differences in coherences usually applied to certain channel x channel comparisons only, and did not encompass brain regions in general. On an individual level, three patients revealed higher coherences between hippocampal and frontal regions during retrieval than during rest (patients 1, 11, and 12). As depicted in the figures, this effect can also be seen for the comparison of re-encoding and resting, however, in fewer channel pairs. In contrast, some patients, especially patient 2, show less ripple-band coherence between hippocampal and frontal areas during retrieval. Interestingly, patient 2 was also the least accurate in the spatial memory task.

In general, coherences are most pronounced between channels within the same regions, and especially within the whole frontal lobe there is high inter-channel coherence in many patients and each analysed frequency band. Coherence results for the theta and gamma band can be found in the Supplementary Material (Figures S1–S4). In other frequencies than the ripple band, there was also no general trend towards an effect of the task conditions on patients’ inter-channel coherences. Mainly patients 1 and 2 revealed different coherences between the conditions; however, these were rather mixed, and no generalized pattern was discernible.

Patient 4, who was most accurate in the spatial memory task, revealed high coherences within the frontal structures across all frequency bands. While this is seen in many patients, the coherence within the frontal regions appears most striking in patient 4. Patient 4 furthermore revealed lower coherences during retrieval as compared to resting within the hippocampal channels, as well as between the parahippocampi and orbitofrontal regions, in the theta band. In other patients, especially the parahippocampal regions did not yield any differences between conditions and in one patient parahippocampal channels even appeared to yield no coherences with other regions at all (patient 9).

In frequency bands other than the ripple-band, most pronounced differences between resting and the conditions are seen in one-third of the patients within the theta band. In the gamma band coherences, especially patients 1 and 2 revealed coherence changes between experimental conditions and resting, but directionalities of these effects were inconsistent across patients. Overall, coherence matrices for the three conditions appear to be very similar for each patient, as can be seen in Figure 3 and Figure 4, as well as in Supplementary Figures S1–S4.

### 3.3. iEEG Coherence Changes over Time

The coherence timelines in the different frequency bands obtained from these analyses were viewed for each electrode pair either as an entire timeline (over the complete recording) or locked to stimuli onsets across averaged retrieval and re-encoding trials. Given the amount of channel x channel combinations, figures were again built on the averages of region-specific channels. The second analysis did not reveal changes in coherences locked to stimuli across retrieval or re-encoding trials for any of the patients. An example figure is provided in the supplementary material (Figure S5).

**Figure 5:**
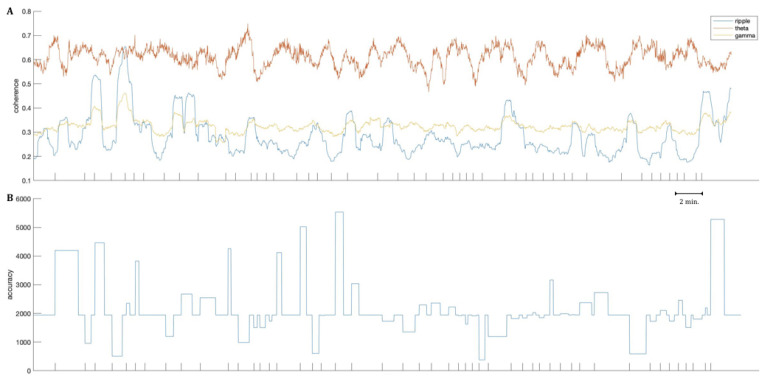
Coherences for patient 9 over the entire recording of 70.57 min within the gamma, theta, and ripple-band. Coherences between channels of the medial-orbitofrontal and the superior-frontal cortex are displayed at the top (**A**). One data point corresponds to a shift of one second, with each coherence value stemming from a moving window of 47 s. At the bottom (**B**), the drop error is depicted for all retrieval trials. Ticks on the abscissa are based on the beginning of each retrieval and re-encoding trial.

Considering coherence changes during the entire recording period, results, however, did reveal short changes in ripple-band coherences for most patients. These changes mainly pertained to interactions involving frontal channels but also the hippocampus. An example is given in Figure 5, showing the coherence changes within the gamma, theta, and ripple-band. Finally, the drop error from the single retrieval trials is plotted at the bottom. Based on the plotted graphs, these changes in ripple-band coherence seem to be unrelated to single trials represented by the x-axis ticks, or the performance accuracy, as changes sometimes lasted for more than one trial and appeared to not be locked to either specifically good or poor performance trials in terms of retrieval accuracy. Eight of our subjects presented such short and rapid changes observable in the timeline plots. In the remaining four, at least hints of these changes could be observed. Further examples are provided within the supplementary material (Figures S6–S10).

Judging from the retrieved timeline figures, ripple-band coherence changes seem to co-occur with changes in the gamma-band coherence, in the example of patient 9. Similar expressions were gained from other patients’ data, however, more subtle, and in no patients were changes in the gamma-band coherence as striking as in the ripple-band. Similarly, the theta-band coherences do not yield as apparent changes, either. The depicted patient is an exception in this regard, as in other patients the gamma-band coherence stays stable over the recording. A further exception to this is presented by patient 12, revealing very rapid and frequent changes in the theta-band coherence, as well (see Figure S10).

## 4. Discussion

The study at hand aimed at investigating the relationship between cognitive processes related to spatial navigation and ripple-band EEG activity. Literature suggests interplay between lower and faster brain oscillations within frontal and hippocampal structures to reflect such processes [34,35,36,54]. We, therefore, investigated frequency band coherence within and between frontal and (para-) hippocampal structures in the intracranial EEG during a virtual navigation task. We neither found a significant increase in ripple occurrence rates from resting to the experimental task conditions, nor did we observe an overall effect of increased coherences within the suspected frequency bands. Thus, the findings do not support the notion of a general effect discernible across the entire patient group enrolled in the experiment.

Regarding the ripple event rates, we suspected an increase in (at least) temporal ripples from resting to both retrieval and re-encoding of objects’ positions within a virtual environment, given the association between HFOs observed in these regions and memory consolidation [14,15]. Surprisingly, we found slightly more ripples in frontal regions in comparison to ripples on spikes, yielding similar occurrence rates overall. Furthermore, ripple rates did not seem to be impacted by the task condition. A reason for both of these observations could lie in physiologic ripples not playing too great a role in retrieval of information but instead in consolidation. This explanation is supported by most studies revealing a connection between memory and physiological ripples in humans during the consolidation phase while subjects were resting [14,19], giving rise to the notion of hippocampal HFOs to reflect spontaneous reactivation that is thought to promote plasticity and stabilize fresh memory contents [55,56]. Contradicting results, however, caution that different memory tasks and recording settings might lead to ripples, both being detected even during the retrieval phase and also being successfully linked to memory performance [57], stressing that complexity and nature of replayed memory contents might affect the neural response during retrieval.

Another explanation for the lack of an increase in ripple rate could be an uneven proportion of artifacts contaminating the signal, with more artifacts during the pre-task period, when subjects did not need to concentrate on the paradigm. Though we did exclude channels that were contaminated with artifacts, we did not perform a general artifacts correction given the nature of the recordings and the findings from the detection algorithm during development. However, more artifacts during the resting period could result in a higher amount of falsely detected ripples due to filtered sharp transients in the process of automatic detection [58,59]. A possible solution would be to manually detect possible ripples. However, manually marking HFOs is problematic in its own respect, as the process is highly subjective and even highly trained EEG specialists seldom consent when it comes to clearly identifying ripples [60]. Furthermore, automated HFO detection becomes necessary when analyzing huge amounts of data simply due to the time-consuming nature of manually detecting HFOs described at length in literature (e.g., Reference [61]).

With regard to the coherence analysis, our findings support isolated task-dependent effects in single subjects, rather than a general effect in coherence changes from resting to experimental conditions across the patient group. There are several possible explanations for our findings. For instance, the selection of reference might constitute a technical issue. Especially, reference channels with high amplitudes were shown to increase correlation between iEEG channels, thus inducing falsely high coherences [62]. Using a common average, however, should prevent this issue as high amplitudes on the reference could only result from a general problem of globally recorded unusually high amplitudes, which seems unlikely given the exclusion of problematic channels before the analysis and the nature of invasive stereo EEG recording. Still, using a common average reference could induce coherences simply by adding a similarity to the signals in the subtraction process [63]. If this induced inter-channel similarity were greater than task-related changes in coherences, a detection of small effects could be prevented. The same problem might occur when using a bipolar-reference, resulting in further similarities on re-referenced channels from the same iEEG electrode, thus increasing possible volume conduction effects. However, Rappelsberger [63] pointed out that an average reference offers some advantages, especially when compared to a common reference.

A further issue stems from the segments we entered into the analysis. The number of segments per patient and condition varied, as well as their length. This is because experimental trials had no fixed duration and for the coherence analysis only segments with long enough duration to reach a sufficient ratio between samples and extracted estimates could be selected [48], being even more severe for high frequencies that require a higher model order for the MVAR model [64]. Since we could only enter sufficiently long segments, we ended up with a selection of long and entire trials only, inducing a possible selection bias. Furthermore, calculating coherences on these whole trials is less sensitive to detecting small and time-restricted changes in frequency-specific coherences, as reported by Raghavachari et al. [65,66]. Investigating changes in theta power and theta connectivity during the course of a working memory task, they found a general increase in theta power but not in coherence during the task [66]. Therefore they suspected that short periods of synchronization had gone amiss due to the measure of coherence over large segments.

This would explain why, whilst finding comparably high theta coherences in general, we did not find effects of changed coherences from resting to retrieval or re-encoding in most patients. In line with our results, suggesting high coherences within specific regions in general, however, with no task-dependent changes over entire periods of encoding and retrieval, scalp EEG studies revealed theta as a rather unstable correlate of memory processes, depending on novelty and repetition effects [67]. Noteworthy is that it has been pointed out that results from scalp EEG studies can differ considerably from findings obtained with intracranial EEG recordings when it comes to theta oscillations and memory [68]. However, the authors also pointed out that the discrepancy might result from iEEG recordings being analyzed using bipolar montages in the majority of studies [68], and there is furthermore no reason to assume that repetition and stimuli parameters differently impact iEEG or scalp EEG-recorded theta activity.

In addition, similar stimuli-dependent impacts might apply to faster frequencies, coupled to slower frequencies [39,67,69,70]. As such, Graetz et al. [67] also found the coupling between gamma and theta oscillations to be unstable. Similar dependences from stimuli parameters have also been reported for the gamma band itself [71], while, in some studies, no correlate of gamma oscillations to cognitive function could be revealed at all [72]. In other examples, gamma oscillations, as well as their coupling with lower frequencies, have further been shown to constitute short periods of synchrony, reflecting early encoding and reactivation within the working memory across different modalities [69,73,74]. Furthermore, the coupling of oscillations has been shown to not uniformly shift in strength during working memory processes, but to heterogeneously strengthen and weaken in time [75].

Taken together, these findings draw a picture of modulatory coupling between frequencies, as well as highly variable involvements of different frequencies, during specific stages of memory processes (i.e., learning, encoding, and retrieval) that speculatively elicit changes in coherences across brain regions for short periods of synchronization only. If this is the case for frequencies up to and including the gamma band, it can also be inferred that similar restrictions apply to the ripple band. This picture might partially explain our results, not showing a general change in coherences, and it is exactly reflected within the results of our additional analysis. Ripple-band coherences appear to increase for short periods throughout the task, but with no obvious relation to single trials, respective conditions, or the performance accuracy. This may further point to a more general activation or reactivation of fronto-temporal networks that is not related to or generalizable across the presentation of stimuli or specific phases of spatial navigation.

Such an interpretation is also backed by the results obtained from the Fourier-spectra comparisons, suggesting no statistically significant differences in power densities between the given task conditions and rest. Furthermore, what becomes apparent from these analyses, is the need to investigate such EEG responses on a single-subject level, as generalizable effects are seldom observed across a patient group and thus might go amiss when conducting group comparisons. This issue is even more problematic in the intracranial EEG, as spatial sampling is incongruent between subjects. One example for this is our finding that parahippocampal regions seem to contribute little to the task-related processes, especially retrieval, as power spectra suggest higher power over all frequencies during resting. However, parahippocampal electrodes had only been recorded in a small portion of patients, and individual results even suggested higher coherences between parahippocampal regions and the hippocampus during retrieval as compared to pre-task resting in one subject.

Another important issue arises from the large width of the ripple band. Averaging coherences from 80 to 249 Hz could cancel out synchronization effects that are isolated to certain frequencies within this band. Although coherences have been reported to be observable over wide frequency ranges (e.g., References [76,77,78]), these studies did not investigate frequency bands quite that large. A possible solution would be to consider smaller frequency bins within the ripple band. However, this would lead to yet another source of variance between the patients, as it is highly unlikely that small effects could be found within the same sub-frequencies across the patient group.

On a final note, the possibility that physiological processes might require or lead to short periods of synchronization within the ripple band instead of generalizable changes occurring over entire epochs of cognition cautions the use of broader measures as exclusive markers for cognition. Similarly, it is questionable whether HFOs as single events can be used as a clinical marker to assess cognitive functioning of brain areas. It has been suggested to use measures derived from higher frequencies within the EEG recordings of patients with epilepsy as a marker to map the eloquent cortex prior to surgery [15]. Our results indicate, however, that different measures, even when derived from one frequency band, can lead to contradicting findings, showcasing the lack in reliability of any given measures in these higher frequencies.

## 5. Conclusions

We were not able to show a general effect of virtual-navigation-related spatial memory performance on iEEG coherences between frontal and (para-)hippocampal regions in any of the observed frequency bands. While some patients elicited trends towards coherence changes from resting to retrieval and re-encoding, these trends varied considerably and do not allow for a well-founded interpretation. These results, however, reflect the debate and uncertainty regarding the specific meaning and function of specific oscillations for memory processes, especially within the higher frequencies (see for example, Reference [79]). Further analysis suggested that rather short periods of interaction within fronto-temporal networks might be at play. The exact timing of these changes, however, is hard to pin down to single events, given the width of segments necessary for calculating coherences derived from these high frequencies. Specifically designed tasks may shed more light on the high-frequency response’s timing within spatial navigation in future works. These short periods, however, may represent a good point to start from in future analysis and suggest high frequencies to be an interesting addition to the common frequency bands for studying cognition.

## Figures and Tables

**Table 1:** Ripple events per second.

Patient #	# ROI Channels	Ripples/Sec. Rest	Ripples/Sec. Retrieval	Ripples/Sec. Re-Encoding	Mean Distance Retrieval
1	7	0.303	0.322	0.198	2612
2	17	0.317	0.324	0.445	3511
3	10	0.352	0.287	0.316	2962
4	13	1.332	1.232	1.614	769
5	64	0.987	1.393	1.211	1266
6	24	0.891	0.705	0.786	2212
7	40	0.857	0.786	0.827	3046
8	26	0.877	1.063	1.006	1774
9	39	0.649	0.588	0.233	1945
10	44	1.175	1.122	1.03	2656
11	23	0.854	0.818	0.793	1082
12	18	0.66	0.712	0.306	1794

**Table 2:** Number of patients with trends of differences (*p* <0.05) in ripple-band coherence between conditions.

			Re-Encoding vs. Resting					
			Hip.	Inf.	Lat.	Med.	Par.	Sup.
**Hippocampus**	4↑ 1↓ (12)		2↑ (12)	2↑ (10)	1↑ (9)	- (8)	1↑ 1↓ (4)	1↑ 1↓ (8)
**Inferior-frontal**	2↑ (10)	2↑ (10)		- (10)	1↑ (7)	1↓ (7)	- (2)	1↑ (7)
**Lat.-orbitofr.**	1↑ 1↓ (9)	1↑ (7)	1↑ (9)		1↑ (9)	- (7)	1↑ (4)	- (7)
**Med.-orbitofr.**	1↑ (8)	- (7)	- (7)	1↑ (8)		- (8)	- (2)	- (8)
**Parahip.**	1↑ 1↓ (4)	1↑ (2)	1↑ (4)	- (2)	1↑ 1↓ (4)		1↑ 1↓ (4)	- (2)
**Superior-fr.**	1↑ 2↓ (8)	1↑ (7)	- (7)	1↓ (8)	- (2)	2↓ (8)		1↑ 2↓ (8)
	**Hip.**	**Inf.**	**Lat.**	**Med.**	**Par.**	**Sup.**		
	**retrieval vs. resting**							

Upward arrows indicate higher coherence during task condition, downward arrows indicated higher coherence during pre-task resting. The number in brackets indicate how many patients had iEEG electrodes within both structures at question. Hip.: Hippocampus; Inf.: Inferior-frontal; Lat./Lat.-orbitofr.: Lateral-orbitofrontal; Med./Med.-orbitofr.: Medial-orbitofrontal; Par./Parahip.: Parahippocampus; Sup./Superior-fr.: Superior-frontal.

## Data Availability

The data presented in this study are available on request from the corresponding author. The data are not publicly available due to privacy reasons.

## Supplementary Materials

The following are available online at https://www.mdpi.com/2076-3425/11/2/162/s1, Figure S1: Coherences for patients 1–6 within the θ band, Figure S2: Coherences for patients 7–12 within the θ band, Figure S3: Coherences for patients 1–6 within the γ band, Figure S4: Coherences for patients 7–12 within the γ band, Figure S5: condition-locked coherences of patient 5, Figure S6: Coherences over time of patient 1, Figure S7: Coherences over time for patient 3, Figure S8: Coherences over time for patient 4, Figure S9: Coherences over time for patient 6, Figure S10: Coherences over time for patient 12.

## Abbreviations

The following abbreviations are used in this manuscript:

iEEG	intracraniel electroencephalography
HFO	high-frequency oscillation

## References

[1] Bragin A., Wilson C.L., Almajano J., Mody I., Engel J. (2004). High-frequency oscillations after status epilepticus: Epileptogenesis and seizure genesis. Epilepsia.

[2] Jacobs J., Zijlmans M., Zelmann R., Chatillon C.É., Hall J., Olivier A., Dubeau F., Gotman J. (2010). High-frequency electroencephalographic oscillations correlate with outcome of epilepsy surgery. Ann. Neurol..

[3] Zijlmans M., Jiruska P., Zelmann R., Leijten F.S., Jefferys J.G., Gotman J. (2012). High-frequency oscillations as a new biomarker in epilepsy. Ann. Neurol..

[4] Frauscher B., Bartolomei F., Kobayashi K., Cimbalnik J., Klooster M.A., Rampp S., Otsubo H., Höller Y., Wu J.Y., Asano E. (2017). High-frequency oscillations: The state of clinical research. Epilepsia.

[5] Bragin A., Engel J., Wilson C.L., Fried I., Buzsáki G. (1999). High-frequency oscillations in human brain. Hippocampus.

[6] Brázdil M., Pail M., Halámek J., Plešinger F., Cimbálník J., Roman R., Klimeš P., Daniel P., Chrastina J., Brichtová E. (2017). Very high frequency oscillations: Novel biomarkers of the epileptogenic zone. Ann. Neurol..

[7] Usui N., Terada K., Baba K., Matsuda K., Usui K., Tottori T., Mihara T., Inoue Y. (2015). Significance of Very-High-Frequency Oscillations (Over 1000 Hz) in Epilepsy. Ann. Neurol..

[8] Höller Y., Kutil R., Klaffenböck L., Thomschewski A., Höller P.M., Bathke A.C., Jacobs J., Taylor A.C., Nardone R., Trinka E. (2015). High-frequency oscillations in epilepsy and surgical outcome. A meta-analysis. Front. Hum. Neurosci..

[9] Cracco R.Q., Cracco J.B. (1976). Somatosensory evoked potential in man: Far field potentials. Clin. Neurophysiol..

[10] Staba R.J., Wilson C.L., Bragin A., Fried I., Engel J. (2002). Quantitative analysis of high-frequency oscillations (80–500 Hz) recorded in human epileptic hippocampus and entorhinal cortex. J. Neurophysiol..

[11] Matsumoto A., Brinkmann B.H., Matthew Stead S., Matsumoto J., Kucewicz M.T., Marsh W.R., Meyer F., Worrell G. (2013). Pathological and physiological high-frequency oscillations in focal human epilepsy. J. Neurophysiol..

[12] Wang S., Wang I.Z., Bulacio J.C., Mosher J.C., Gonzalez-Martinez J., Alexopoulos A.V., Najm I.M., So N.K. (2013). Ripple classification helps to localize the seizure-onset zone in neocortical epilepsy. Epilepsia.

[13] Alkawadri R., Gaspard N., Goncharova I.I., Spencer D.D., Gerrard J.L., Zaveri H., Duckrow R.B., Blumenfeld H., Hirsch L.J. (2014). The spatial and signal characteristics of physiologic high frequency oscillations. Epilepsia.

[14] Axmacher N., Elger C.E., Fell J. (2008). Ripples in the medial temporal lobe are relevant for human memory consolidation. Brain.

[15] Kunii N., Kawai K., Kamada K., Ota T., Saito N. (2014). The significance of parahippocampal high gamma activity for memory preservation in surgical treatment of atypical temporal lobe epilepsy. Epilepsia.

[16] Chrobak J.J., Buzsáki G. (1996). High-Frequency Oscillations in the Output Networks of the Hippocampal-Entorhinal Axis of the Freely Behaving Rat. J. Neurosci..

[17] Fernández-Ruiz A., Oliva A., de Oliveira E.F., Rocha-Almeida F., Tingley D., Buzsáki G. (2019). Long-duration hippocampal sharp wave ripples improve memory. Science.

[18] Wilson M.A., McNaughton B.L. (1994). Reactivation of hippocampal ensemble memories during sleep. Science.

[19] Pu Y., Cornwell B.R., Cheyne D., Johnson B.W. (2018). High-gamma activity in the human hippocampus and parahippocampus during inter-trial rest periods of a virtual navigation task. NeuroImage.

[20] Jacobs J., Banks S., Zelmann R., Zijlmans M., Jones-Gotman M., Gotman J. (2016). Spontaneous ripples in the hippocampus correlate with epileptogenicity and not memory function in patients with refractory epilepsy. Epilepsy Behav..

[21] Thomschewski A., Hincapié A.S., Frauscher B. (2019). Localization of the Epileptogenic Zone Using High Frequency Oscillations. Front. Neurol..

[22] Binnie C., Trenite D.K.N., Smit A., Wilkins A. (1987). Interactions of epileptiform EEG discharges and cognition. Epilepsy Res..

[23] Holmes G.L., Lenck-Santini P.P. (2006). Role of interictal epileptiform abnormalities in cognitive impairment. Epilepsy Behav..

[24] Kleen J.K., Scott R.C., Holmes G.L., Roberts D.W., Rundle M.M., Testorf M., Lenck-Santini P.P., Jobst B.C. (2013). Hippocampal interictal epileptiform activity disrupts cognition in humans. Neurology.

[25] Melani F., Zelmann R., Mari F., Gotman J. (2013). Continuous high frequency activity: A peculiar SEEG pattern related to specific brain regions. Clin. Neurophysiol..

[26] Liu S., Gurses C., Sha Z., Quach M.M., Sencer A., Bebek N., Curry D.J., Prabhu S., Tummala S., Henry T.R. (2018). Stereotyped high-frequency oscillations discriminate seizure onset zones and critical functional cortex in focal epilepsy. Brain.

[27] Vaz A.P., Inati S.K., Brunel N., Zaghloul K.A. (2019). Coupled ripple oscillations between the medial temporal lobe and neocortex retrieve human memory. Science.

[28] Buzsáki G., Moser E.I. (2013). Memory, navigation and theta rhythm in the hippocampal-entorhinal system. Nat. Neurosci..

[29] O’Keefe J., Burgess N. (1999). Theta activity, virtual navigation and the human hippocampus. Trends Cogn. Sci..

[30] Watrous A.J., Lee D.J., Izadi A., Gurkoff G.G., Shahlaie K., Ekstrom A.D. (2013). A comparative study of human and rat hippocampal low-frequency oscillations during spatial navigation. Hippocampus.

[31] McNab F., Klingberg T. (2008). Prefrontal cortex and basal ganglia control access to working memory. Nat. Neurosci..

[32] Kaplan R., Bush D., Bonnefond M., Bandettini P.A., Barnes G.R., Doeller C.F., Burgess N. (2014). Medial prefrontal theta phase coupling during spatial memory retrieval. Hippocampus.

[33] Sharma G., Gramann K., Chandra S., Singh V., Mittal A.P. (2017). Brain connectivity during encoding and retrieval of spatial information: Individual differences in navigation skills. Brain Inform..

[34] Hyman J.M., Zilli E.A., Paley A.M., Hasselmo M.E. (2005). Medial prefrontal cortex cells show dynamic modulation with the hippocampal theta rhythm dependent on behavior. Hippocampus.

[35] Siapas A.G., Lubenov E.V., Wilson M.A. (2005). Prefrontal phase locking to hippocampal theta oscillations. Neuron.

[36] Negrón-Oyarzo I., Espinosa N., Aguilar M., Fuenzalida M., Aboitiz F., Fuentealba P. (2018). Coordinated prefrontal–hippocampal activity and navigation strategy-related prefrontal firing during spatial memory formation. Proc. Natl. Acad. Sci. USA.

[37] Araújo D.B.d., Baffa O., Wakai R.T. (2002). Theta oscillations and human navigation: A magnetoencephalography study. J. Cogn. Neurosci..

[38] Bischof W.F., Boulanger P. (2003). Spatial navigation in virtual reality environments: An EEG analysis. CyberPsychol. Behav..

[39] Alekseichuk I., Turi Z., de Lara G.A., Antal A., Paulus W. (2016). Spatial working memory in humans depends on theta and high gamma synchronization in the prefrontal cortex. Curr. Biol..

[40] Doeller C.F., Barry C., Burgess N. (2010). Evidence for grid cells in a human memory network. Nature.

[41] Chen D., Kunz L., Wang W., Zhang H., Wang W.X., Schulze-Bonhage A., Reinacher P.C., Zhou W., Liang S., Axmacher N. (2018). Hexadirectional modulation of theta power in human entorhinal cortex during spatial navigation. Curr. Biol..

[42] Kunz L., Schröder T.N., Lee H., Montag C., Lachmann B., Sariyska R., Reuter M., Stirnberg R., Stöcker T., Messing-Floeter P.C. (2015). Reduced grid-cell-like representations in adults at genetic risk for Alzheimer?s disease. Science.

[43] Kunz L., Wang L., Lachner-Piza D., Zhang H., Brandt A., Dümpelmann M., Reinacher P.C., Coenen V.A., Chen D., Wang W.X. (2019). Hippocampal theta phases organize the reactivation of large-scale electrophysiological representations during goal-directed navigation. Sci. Adv..

[44] Qin C., Tan Z., Pan Y., Li Y., Wang L., Ren L., Zhou W., Wang L. (2017). Automatic and precise localization and cortical labeling of subdural and depth intracranial electrodes. Front. Neuroinform..

[45] Park J., Lee H., Kim T., Park G.Y., Lee E.M., Baek S., Ku J., Kim I.Y., Kim S.I., Jang D.P. (2014). Role of low-and high-frequency oscillations in the human hippocampus for encoding environmental novelty during a spatial navigation task. Hippocampus.

[46] Lachner-Piza D., Jacobs J., Bruder J.C., Schulze-Bonhage A., Stieglitz T., Duempelmann M. (2020). Automatic detection of high-frequency-oscillations and their sub-groups co-occurring with interictal-epileptic-spikes. J. Neural Eng..

[47] Schlögl A., Brunner C. (2008). BioSig: A free and open source software library for BCI research. Computer.

[48] Kus R., Kaminski M., Blinowska K.J. (2004). Determination of EEG activity propagation: Pair-wise versus multichannel estimate. IEEE Trans. Biomed. Eng..

[49] Nolte G., Bai O., Wheaton L., Mari Z., Vorbach S., Hallett M. (2004). Identifying true brain interaction from EEG data using the imaginary part of coherency. Clin. Neurophysiol..

[50] Höller Y., Butz K., Thomschewski A., Schmid E., Uhl A., Bathke A.C., Zimmermann G., Tomasi S.O., Nardone R., Staffen W. (2017). Reliability of EEG interactions differs between measures and is specific for neurological diseases. Front. Hum. Neurosci..

[51] Duffy F.H., Iyer V.G., Surwillo W.W. (1989). Clinical Electroencephalography and Topographic Brain Mapping: Technology and Practice.

[52] Maris E., Oostenveld R. (2007). Nonparametric statistical testing of EEG- and MEG-data. J. Neurosci. Methods.

[53] Dunn O.J. (1961). Multiple comparisons among means. J. Am. Stat. Assoc..

[54] Axmacher N., Henseler M.M., Jensen O., Weinreich I., Elger C.E., Fell J. (2010). Cross-frequency coupling supports multi-item working memory in the human hippocampus. Proc. Natl. Acad. Sci. USA.

[55] Roumis D.K., Frank L.M. (2015). Hippocampal sharp-wave ripples in waking and sleeping states. Curr. Opin. Neurobiol..

[56] Buzsáki G. (2015). Hippocampal sharp wave-ripple: A cognitive biomarker for episodic memory and planning. Hippocampus.

[57] Vaz A.P., Wittig J.H., Inati S.K., Zaghloul K.A. (2020). Replay of cortical spiking sequences during human memory retrieval. Science.

[58] Reva N., Aftanas L. (2004). The coincidence between late non-phase-locked gamma synchronization response and saccadic eye movements. Int. J. Psychophysiol..

[59] Yuval-Greenberg S., Tomer O., Keren A.S., Nelken I., Deouell L.Y. (2008). Transient induced gamma-band response in EEG as a manifestation of miniature saccades. Neuron.

[60] Spring A.M., Pittman D.J., Aghakhani Y., Jirsch J., Pillay N., Bello-Espinosa L.E., Josephson C., Federico P. (2017). Interrater reliability of visually evaluated high frequency oscillations. Clin. Neurophysiol..

[61] von Ellenrieder N., Andrade-Valença L.P., Dubeau F., Gotman J. (2012). Automatic detection of fast oscillations (40–200 Hz) in scalp EEG recordings. Clin. Neurophysiol..

[62] Hu S., Stead M., Dai Q., Worrell G.A. (2010). On the recording reference contribution to EEG correlation, phase synchorony, and coherence. IEEE Trans. Syst. Man Cybern. Part B (Cybern.).

[63] Rappelsberger P. (1989). The reference problem and mapping of coherence: A simulation study. Brain Topogr..

[64] Schlögl A., Supp G. (2006). Analyzing event-related EEG data with multivariate autoregressive parameters. Prog. Brain Res..

[65] Raghavachari S., Kahana M.J., Rizzuto D.S., Caplan J.B., Kirschen M.P., Bourgeois B., Madsen J.R., Lisman J.E. (2001). Gating of human theta oscillations by a working memory task. J. Neurosci..

[66] Raghavachari S., Lisman J.E., Tully M., Madsen J.R., Bromfield E., Kahana M.J. (2006). Theta oscillations in human cortex during a working-memory task: Evidence for local generators. J. Neurophysiol..

[67] Graetz S., Daume J., Friese U., Gruber T. (2019). Alterations in oscillatory cortical activity indicate changes in mnemonic processing during continuous item recognition. Exp. Brain Res..

[68] Herweg N.A., Solomon E.A., Kahana M.J. (2020). Theta oscillations in human memory. Trends Cogn. Sci..

[69] Barnes J.J., Nobre A.C., Woolrich M.W., Baker K., Astle D.E. (2016). Training working memory in childhood enhances coupling between frontoparietal control network and task-related regions. J. Neurosci..

[70] Berger B., Griesmayr B., Minarik T., Biel A., Pinal D., Sterr A., Sauseng P. (2019). Dynamic regulation of interregional cortical communication by slow brain oscillations during working memory. Nat. Commun..

[71] Busch N.A., Debener S., Kranczioch C., Engel A.K., Herrmann C.S. (2004). Size matters: Effects of stimulus size, duration and eccentricity on the visual gamma-band response. Clin. Neurophysiol..

[72] Juergens E., Guettler A., Eckhorn R. (1999). Visual stimulation elicits locked and induced gamma oscillations in monkey intracortical-and EEG-potentials, but not in human EEG. Exp. Brain Res..

[73] van Vugt M.K., Chakravarthi R., Lachaux J.P. (2014). For whom the bell tolls: Periodic reactivation of sensory cortex in the gamma band as a substrate of visual working memory maintenance. Front. Hum. Neurosci..

[74] Tseng Y.L., Liu H.H., Liou M., Tsai A.C., Chien V.S., Shyu S.T., Yang Z.S. (2019). Lingering Sound: Event-Related Phase-Amplitude Coupling and Phase-Locking in Fronto-Temporo-Parietal Functional Networks During Memory Retrieval of Music Melodies. Front. Hum. Neurosci..

[75] Maris E., van Vugt M., Kahana M. (2011). Spatially distributed patterns of oscillatory coupling between high-frequency amplitudes and low-frequency phases in human iEEG. Neuroimage.

[76] Sarnthein J., Petsche H., Rappelsberger P., Shaw G., Von Stein A. (1998). Synchronization between prefrontal and posterior association cortex during human working memory. Proc. Natl. Acad. Sci. USA.

[77] von Stein A., Rappelsberger P., Sarnthein J., Petsche H. (1999). Synchronization between temporal and parietal cortex during multimodal object processing in man. Cereb. Cortex.

[78] von Stein A., Sarnthein J. (2000). Different frequencies for different scales of cortical integration: From local gamma to long range alpha/theta synchronization. Int. J. Psychophysiol..

[79] Burke J.F., Ramayya A.G., Kahana M.J. (2015). Human intracranial high-frequency activity during memory processing: Neural oscillations or stochastic volatility?. Curr. Opin. Neurobiol..

