# Supplementary material for: Temporo-Frontal Coherences and High-Frequency iEEG Responses during Spatial Navigation in Patients with Drug-Resistant Epilepsy
Source: Brain Sci. 2021 Jan 26;11(2):162. doi: 10.3390/brainsci11020162 (PMC7911024; doi:10.3390/brainsci11020162)
Supplement: Supplementary file 1 [file brainsci-11-00162-s001.pdf]

Supplementary Materials: TEMPORO-FRONTAL COHERENCES AND HIGH-FREQUENCY iEEG RESPONSES DURING SPATIAL NAVIGATION IN PATIENTS WITH DRUG-RESISTANT EPILEPSY

Aljoscha Thomschewski [1,2*], Eugen Trinka [1] and Julia Jacobs[3,4,5]

1. iEEG coherence results I

Depicted are absolute coherence values in channel x channel matrices for both experimental conditions as well as resting (columns 1-3). Column 4 shows the respective contrasts between retrieval versus resting in the bottom left triangle under the diagonal and reencoding versus rest in the upper right triangle. Red (positive) p-values represent higher connectivity in the experimental condition and blue (negative) values represent higher connectivity during the resting. Supplementary figures include coherence matrices for all patients in the θ (4-7 Hz) and γ frequency bands (40-79 Hz). For each frequency band, there are two figures, with the first depicting the results for patients one to six and the second showing the connectivity matrices for patients seven to twelve. Brain regions of interest are abbreviated as follows: Hip = hippocampus, Inf = inferior-frontal , Lat = lateral-orbitofrontal, Med = medial-orbitofrontal, Par = parahippocampus, Sup = superior-frontal.

2. iEEG coherence results II

Depicted are coherences over time derived from autoregressive models calculated for segments of 47 seconds each, with a moving window of one second. One data point corresponds to a shift of one second. In the first figure, an example of trial locked coherences is presented, that is coherences were averaged according to the beginning of retrieval and re-encoding trials, and then grouped for bad and good trials via median-split (Figure S5). Figures S6 to S10 depict coherences over time for single patients and chosen region-to-region interactions. On the bottom of each figure, the drop error is depicted for all retrieval trials. Ticks on the abscissae are based on the beginning of each retrieval and re-encoding trial.

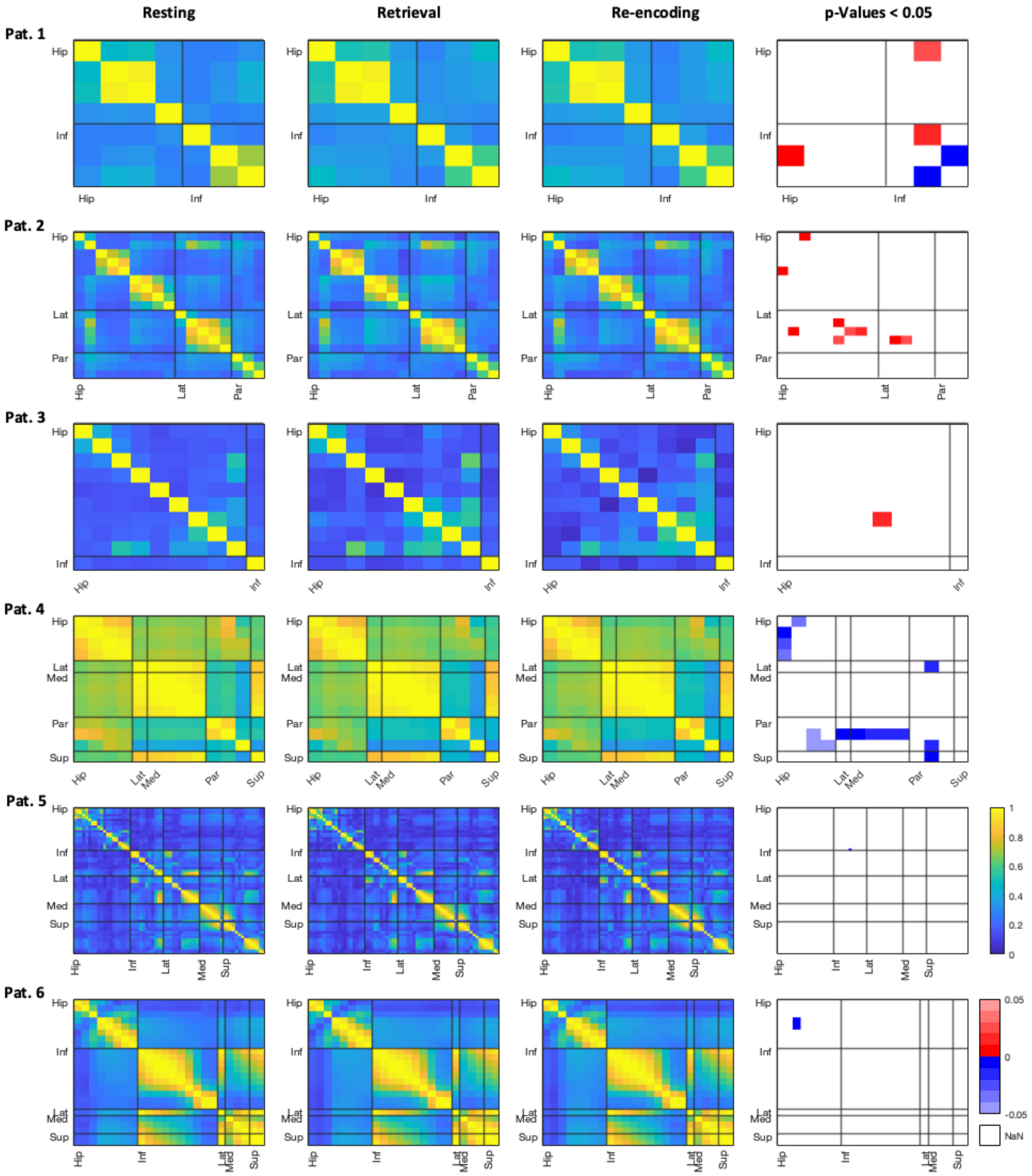

Figure S1. Coherences for patients 1-6 within the θ band.

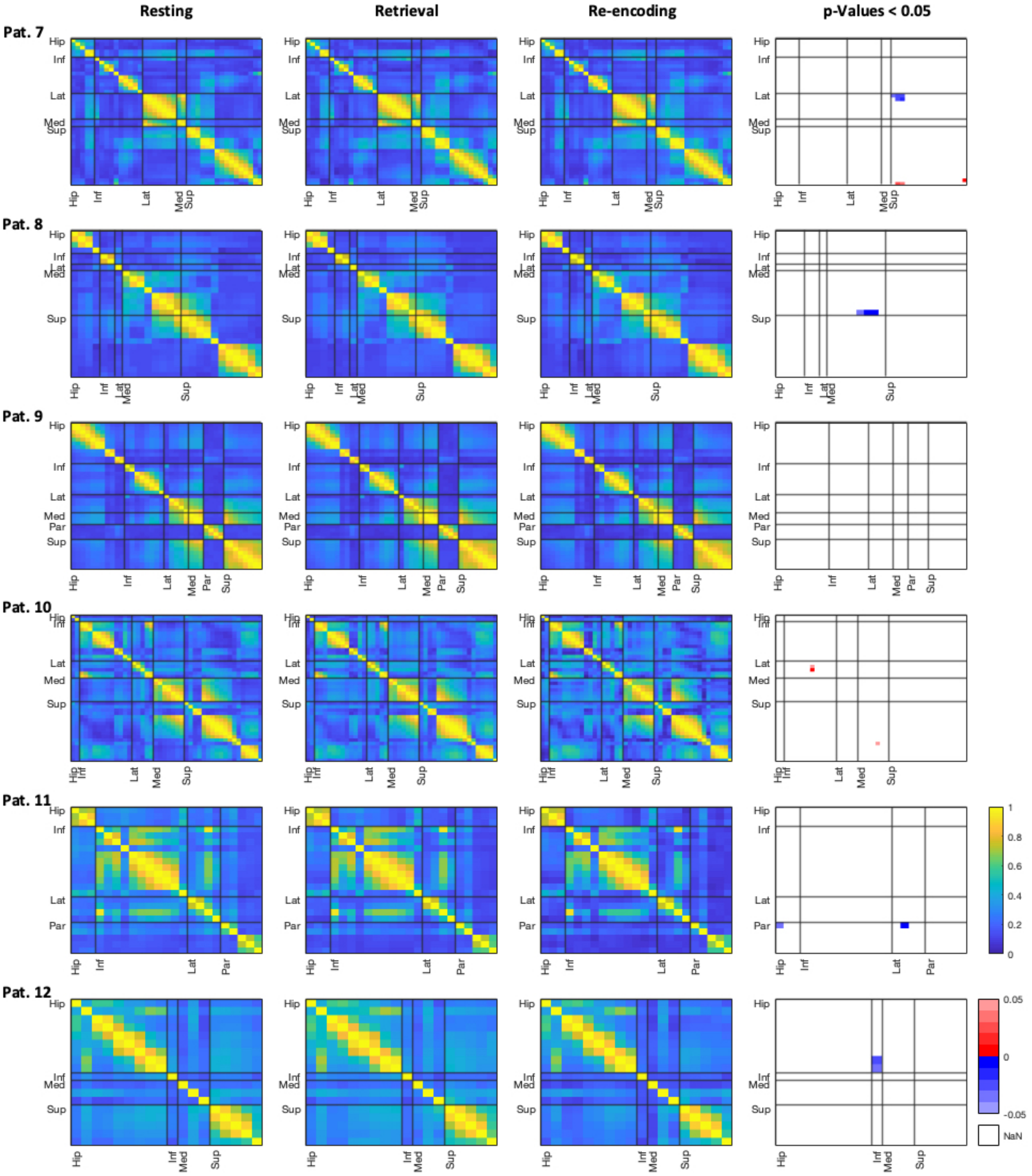

Figure S2. Coherences for patients 7-12 within the θ band.

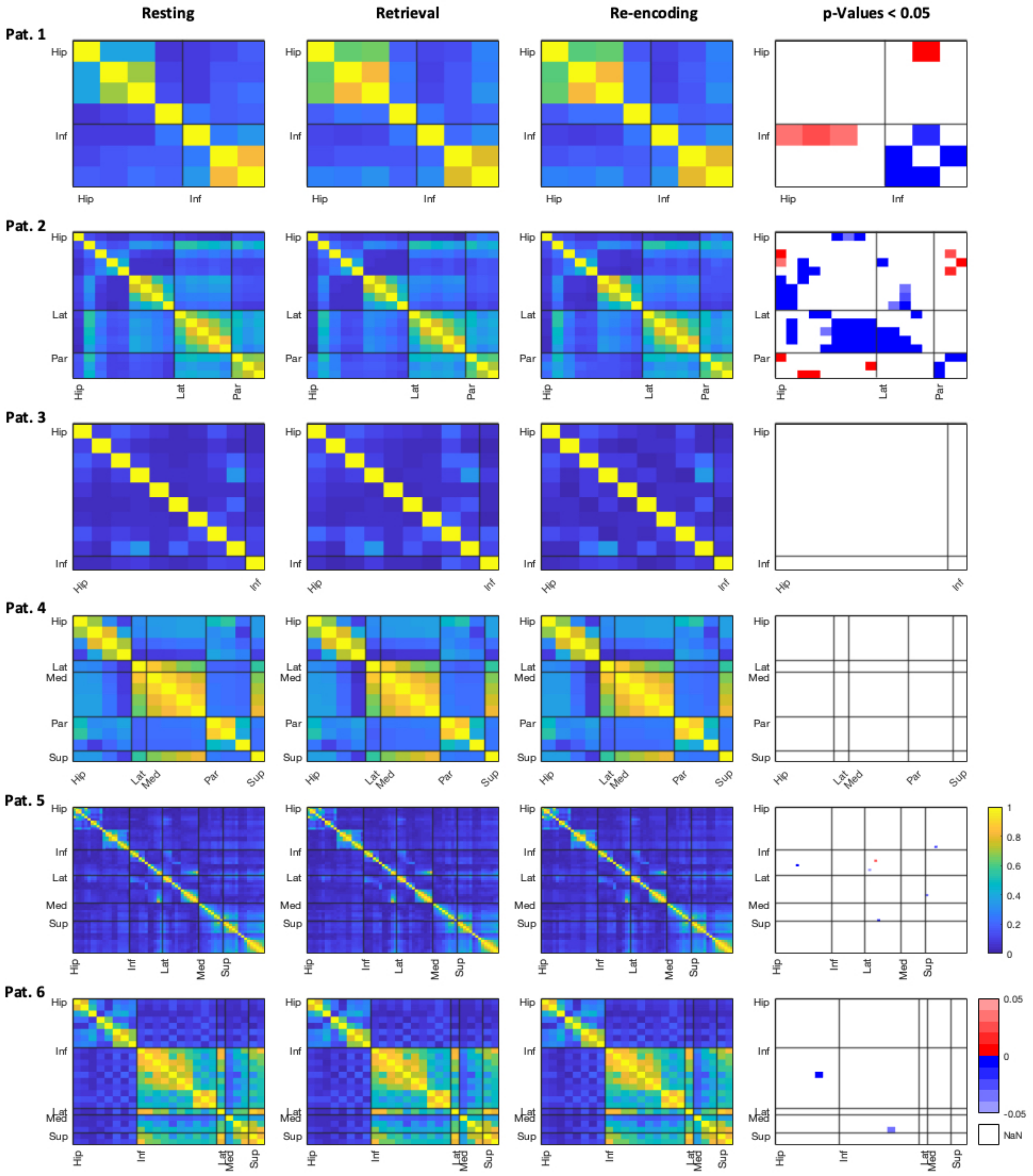

Figure S3. Coherences for patients 1-6 within the γ band.

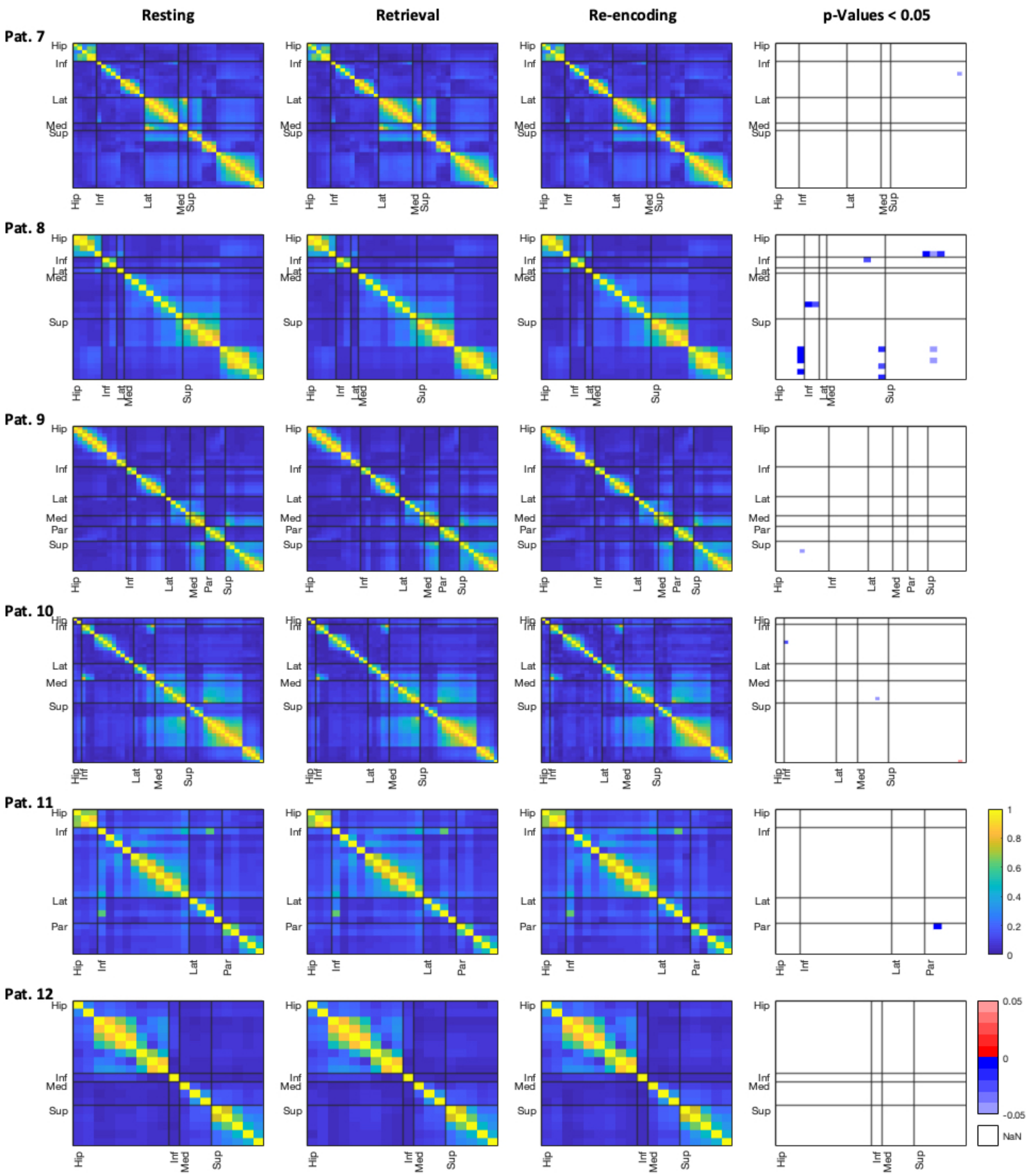

Figure S4. Coherences for patients 7-12 within the γ band.

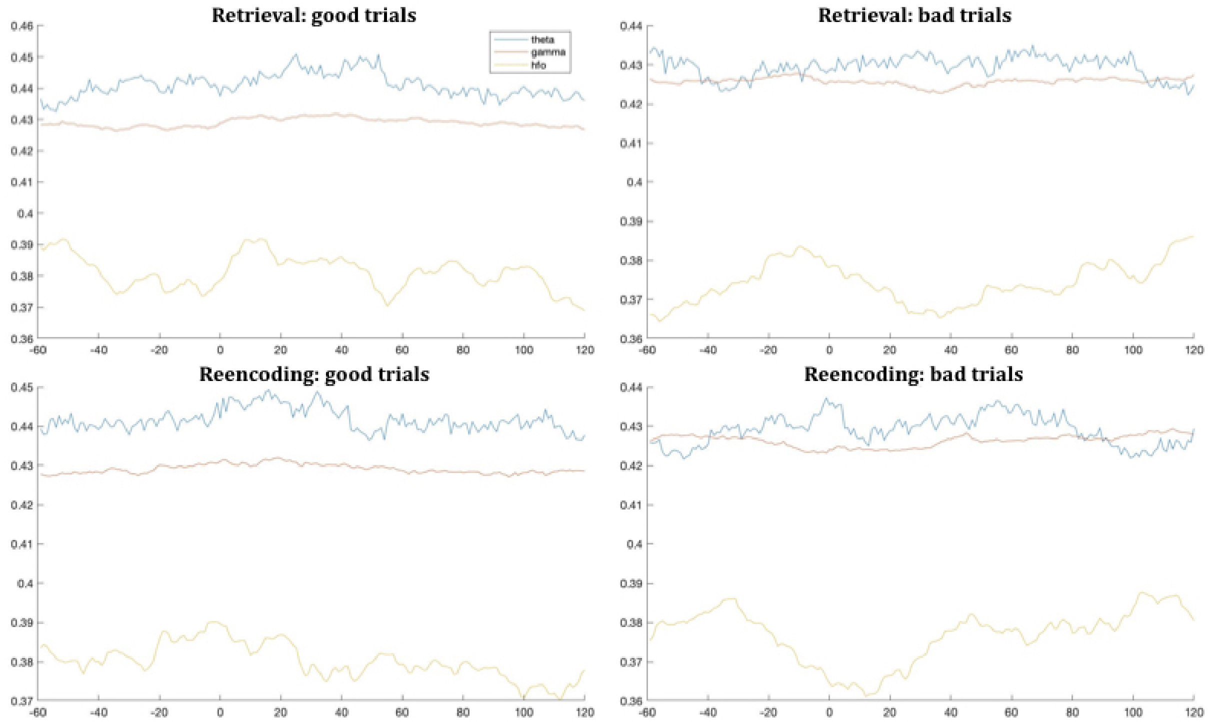

Figure S5. Condition-locked coherences of patient 5 from 60 second before condition onset to two minutes after.

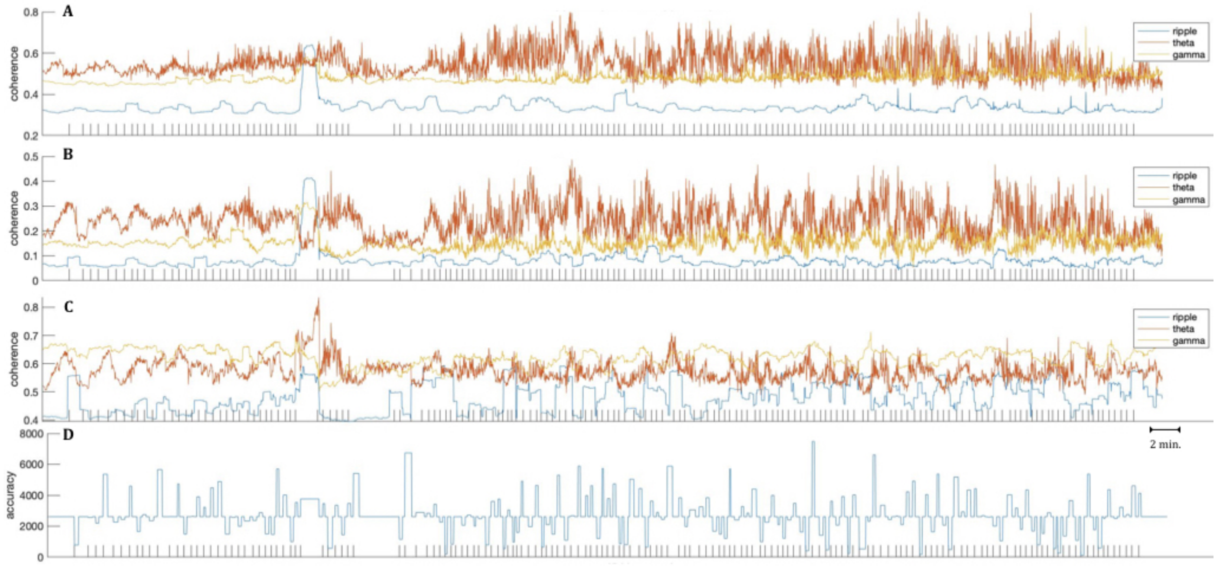

Figure S6. Coherences over time of patient 1 for channel interactions within the hippocampus (A), between the hippocampus and inferior-frontal regions (B), and within the inferior frontal lobe (C). Drop errors for the retrieval trials are plotted at the bottom (D).

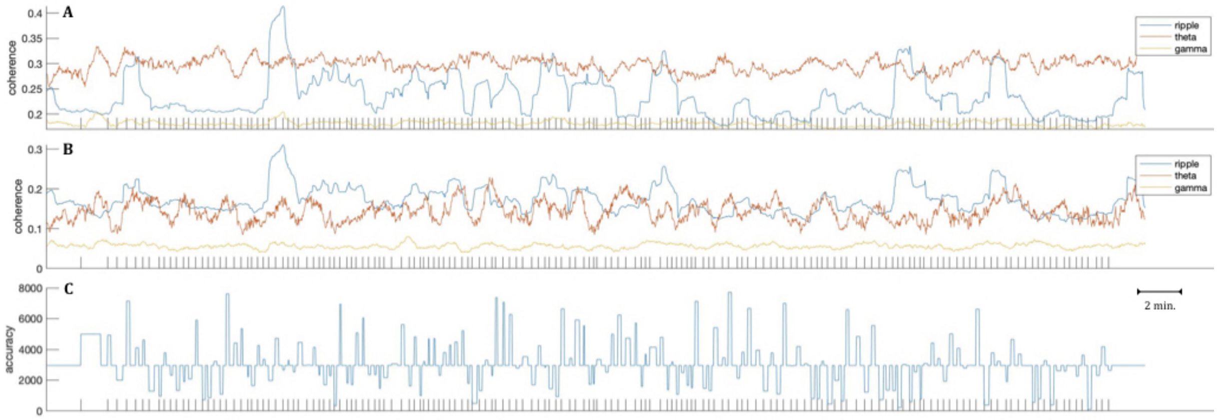

Figure S7. Coherences over time of patient 3 for channel interactions within the hippocampus (A), and between the hippocampus and inferior-frontal regions (B). Drop errors for the retrieval trials are plotted at the bottom (C).

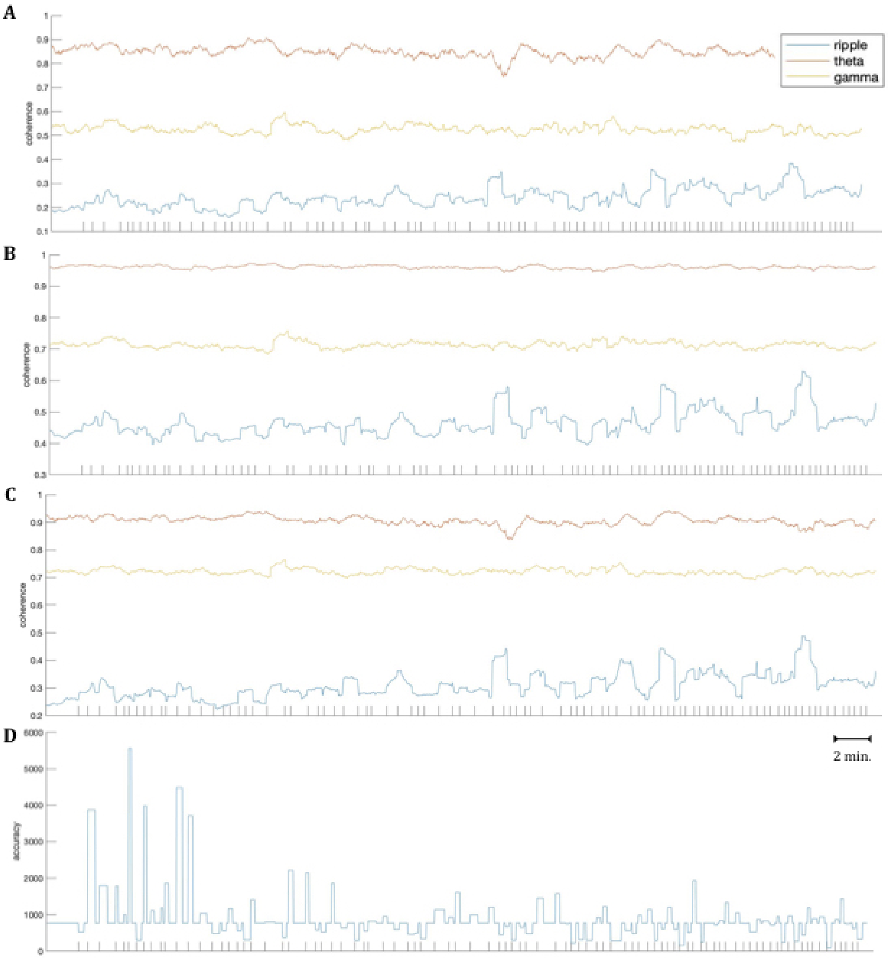

Figure S8. Coherences over time of patient 4 for channel interactions between the lateral-orbitofrontal cortex and superior-frontal regions (A) as well as the medial-orbitofrotal cortex (B). Interactions between channels of the medial-orbitofrontal and the superior-frontal cortices are shown in (C), and the drop errors for the retrieval trials are plotted at the bottom (D).

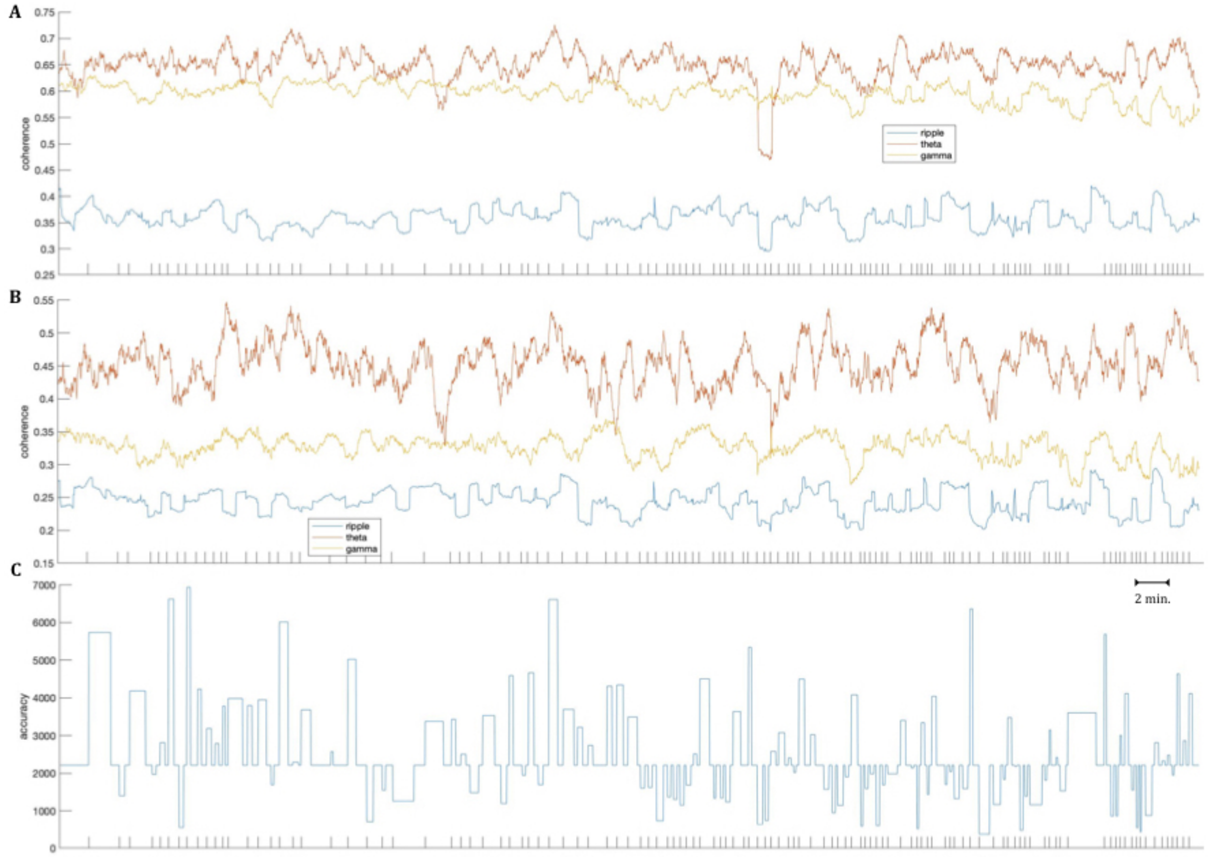

Figure S9. Coherences over time of patient 6 for channel interactions between the inferior-frontal cortex and the lateral-orbitofrontal cortex (A) as well as the medial-orbitofrontal cortex (B). Drop errors for the retrieval trials are plotted at the bottom (C).

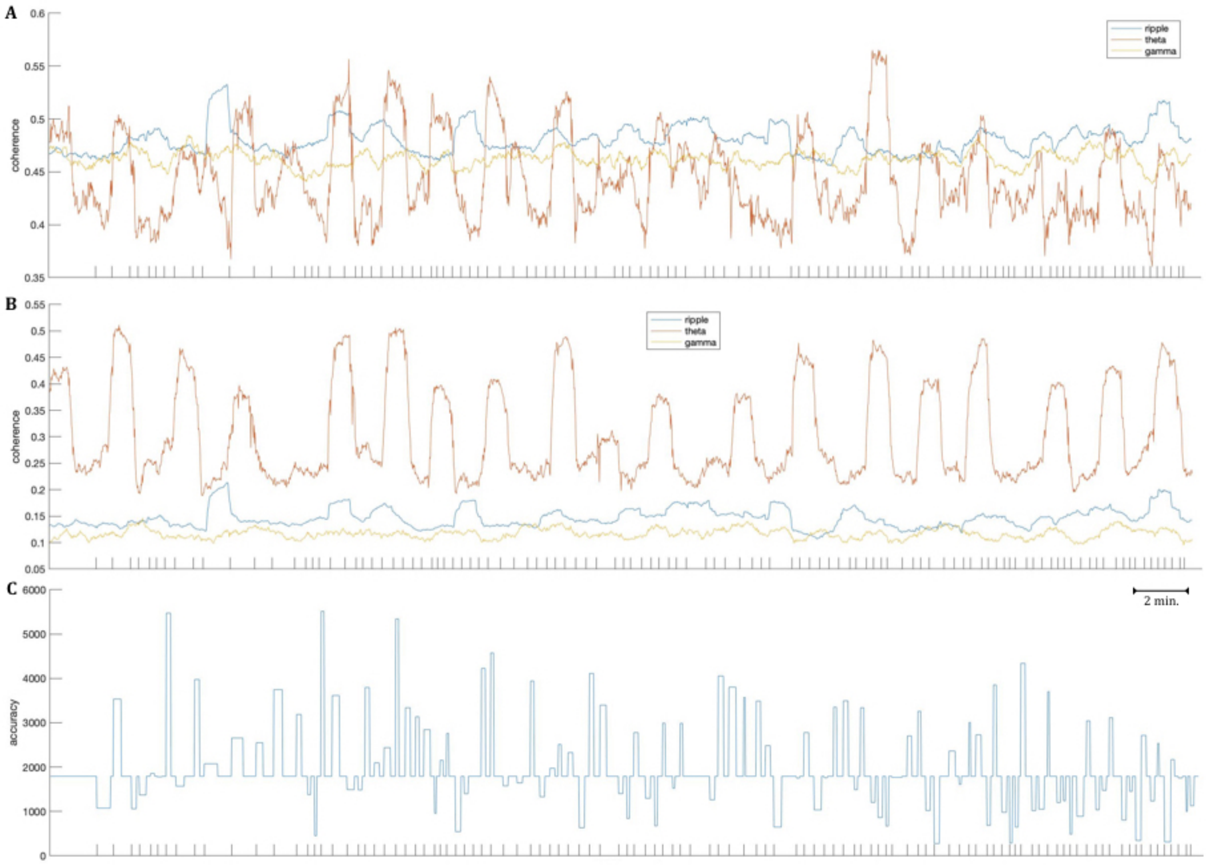

Figure S10. Coherences over time of patient 12 for channel interactions within the medial-orbitofrontal cortex (A), and between the medial-orbitofrontal and the superior-frontal cortex (B). Drop errors for the retrieval trials are plotted at the bottom (C).
